# Building an Ensemble of Fine-Tuned Naive Bayesian Classifiers for Text Classification

**DOI:** 10.3390/e20110857

**Published:** 2018-11-07

**Authors:** Khalil El Hindi, Hussien AlSalman, Safwan Qasem, Saad Al Ahmadi

**Affiliations:** Department of Computer Science, College of Computer and Information Sciences, King Saud University, Riyadh 11543, Saudi Arabia

**Keywords:** text classification, ensembles of classifiers, naive Bayesian learning, fine-tuning naive Bayesian algorithm, machine learning

## Abstract

Text classification is one domain in which the naive Bayesian (NB) learning algorithm performs remarkably well. However, making further improvement in performance using ensemble-building techniques proved to be a challenge because NB is a stable algorithm. This work shows that, while an ensemble of NB classifiers achieves little or no improvement in terms of classification accuracy, an ensemble of fine-tuned NB classifiers can achieve a remarkable improvement in accuracy. We propose a fine-tuning algorithm for text classification that is both more accurate and less stable than the NB algorithm and the fine-tuning NB (FTNB) algorithm. This improvement makes it more suitable than the FTNB algorithm for building ensembles of classifiers using bagging. Our empirical experiments, using 16-benchmark text-classification data sets, show significant improvement for most data sets.

## 1. Introduction

In text classification, the task is to assign a document to a category of a predefined set of categories. It has many real-world applications, including information retrieval, spam filtering [1], email routing [2], sentiment analysis, and many other automated document processing applications. Text classification is also a challenging classification problem for several reasons [3,4]: A typical text classification data set consists of thousands of features, many of them redundant, which may cause overfitting and may violate the conditional independence assumption of NB. In addition, the data sets are likely to be imbalanced in the sense that the number of positive documents may be much smaller than the number of negative documents. 

Building an ensemble of classifiers is a widely-used method to improve the accuracy of machine learning methods. Bagging [5] and boosting [6,7] are probably the most widely used methods for building ensembles of classifiers. They train the constituent classifiers using different samples of the training data. Different samples of training data are used to make sure that the classifiers are diverse because it would be meaningless to combine several classifiers that make the same predictions.

The naive Bayesian learning algorithm performs remarkably well for text classification [8,9,10,11]. However, making further improvement by building an ensemble of several NB classifiers is a challenge because NB is a stable algorithm [12], in the sense that a small change in the training data does not lead to a substantially different classifier. This has the advantage of making it robust to noisy data [13] but, at the same time, limits the improvements that can be achieved from building an ensemble of NB classifiers using bagging or boosting [7]. In [12], it was argued that, due to its stability, little or no improvement can be achieved by building an ensemble of NB classifiers using the AdaBoost algorithm [7].

In [14], a fine-tuning algorithm for NB classifiers (FTNB) was introduced. Although the algorithm significantly improves the classification accuracy of the NB algorithm in many application domains, the algorithm sacrifices the noise tolerance advantage of the NB algorithm [15]. This indicates that FTNB is less stable than NB and may, therefore, be more suitable for building an ensemble of classifiers than the NB algorithm. Moreover, the FTNB algorithm uses a learning rate parameter that can be set to different values to increase the diversity of classifiers. 

In this work, we use the fine-tuning method to generate a diverse ensemble of NB classifiers for text classification. We also modify the fine-tuning algorithm to make it less stable and thus produce more diverse classifiers. The modifications also make the algorithm more accurate for text classification. We use Breiman’s bagging method [5] to build the ensembles. 

The work is structured as follows: In Section 2, we review the related work on text classification and building ensembles of classifiers. In Section 3, we review the FTNB algorithm and propose some modifications. In Section 4, we present the results we obtained from bagging the NB, the FTNB, and the modified FTNB algorithms. Section 5 is the conclusion.

## 2. Related Work

This section is divided into two subsections: In the first, we review the related work on ensembles of classifiers in general, and building ensembles of NB classifiers in particular; in the second, we review the FTNB algorithm [14] for fine-tuning NB classifiers.

### 2.1. Building Ensembles of Classifiers

Building ensembles of classifiers has been an active research area since 1990s [16]. Entire books [16,17,18,19], have been devoted to the subject, which reflects the continuing interest in this field. Building ensembles of classifiers is widely used to enhance the classification accuracy of machine learning algorithms. The basic idea is to build an ensemble of classifiers that collectively gives better classification accuracy than any of the constituent classifiers. However, there are two conditions that must be satisfied for the ensemble to be more accurate than its constituent classifiers [20]: First, the error rate of each individual classifier must be less than 50%; second, the classifiers must be diverse. Diversity is a key and challenging issue [16], because it would be meaningless to combine several classifiers that make the same predictions. Diversity can be achieved in different ways, and perhaps the most widely used method is to build classifiers using different samples of the training data, which is usually done either by bagging [5] or boosting [6]. 

Bagging draws a random sample of the training set, and uses it to construct a constituent classifier. The sample is of the same size as the training set and, as a result, many of the training instances may occur more than once in a sample. Bagging uses a uniform probability distribution to construct each sample, giving all instances an equal probability of being selected. Boosting, on the other hand, uses a probability distribution in sampling that favors the instances that were misclassified by previous classifiers.

Bagging and Boosting work well with learning algorithms that are not stable [16]. A learning algorithm is unstable if a small change in the training data produces a substantially different classifier. Diversity can also be achieved by using different learning parameters [21], such as the initial weights of connections in artificial neural networks. Other methods achieve diversity by using different features to build classifiers [22].

The NB algorithm is known to be a stable classifier [12]. Though stability makes the NB algorithm robust to noise [13], it also makes it difficult to construct an ensemble of NB classifiers using an ensemble building method that relies on data sample manipulation, such as bagging and boosting. This is actually the case with any stable classifier [16]. The reason behind it is the fact that slightly different data samples do not cause a base learner to generate sufficiently diverse classifiers [16]. In [12], it was shown that little or no improvement can be achieved by building an ensemble of NB classifiers using the AdaBoost algorithm, and it was suggested that a tree structure be built into the NB algorithm. In [23], it was reported that, unlike the case of neural network and decision tree classifiers, bagging NB classifiers did not reduce the classification error for morphological galaxy classification. In an attempt to increase the diversity of NB classifiers, [24] generalizes the random forest (RF) approach [25] to NB. The RF approach for building an ensemble of classifiers uses resampling by bagging and a random set of features to build each classifier. However, [24] reports a slight increase in the accuracy of NB as a result of generalizing RF to NB.In [26], an ensemble of heterogeneous Bayesian network classifiers were proposed, diversity was mainly achieved by building ensembles of Bayesian classifiers of different structures. 

### 2.2. Fine-Tuning the NB Algorithm 

The NB algorithm uses Bayes’ conditional probability rule for classifying instances. To classify an instance of the form $<a_{1},a_{2},\cdots ,a_{m}>$, Bayes’ rule (Equation (1)) is used to find the class that has the maximum probability given the instance’s attribute values,

(1) $$class=\underset{c\in C}{\mathrm{argmax}}\frac{p(a_{1},a_{2},\cdots ,a_{m}|c)\cdot p\left(c\right)}{p\left(a_{1},a_{2},\ldots ,a_{m}\right)},$$

where C is a vector of all class attribute values. p(c) is the probability of class *c*. $p\left(a_{1},a_{2},\ldots ,a_{m}\right)\;$ is the probability that attributes 1, 2, …, *m* will take the values $a_{1},a_{2},\cdots ,a_{m}$, respectively. $p(a_{1},a_{2},\cdots ,a_{m}|c)$ is the probability that attributes 1, 2, …, *m* will take the values $a_{1},a_{2},\cdots ,a_{m}$, given that the instance is of class *c*.

The algorithm makes the naive assumption that all attribute values are conditionally independent given the class values; therefore,

(2) $$p\left(a_{1},a_{2},\cdots ,a_{m}|c\right)=\underset{j}{\prod }p(a_{j}|c),$$

Additionally, because, given a certain instance, the denominator $p\left(a_{1},a_{2},\ldots ,a_{m}\right)$ is the same for all classes, Equation (1) can be simplified as

(3) $$class=\underset{c\in C}{\mathrm{argmax}\;}p\left(c\right)\cdot \underset{j}{\prod }p(a_{j}|c),$$

Clearly, the accuracy of the NB algorithm depends on finding accurate estimates for the probability terms p(c) and $p\left(a_{j}|c\right)$, which are estimated using the available training data. This could be challenging, especially in domains with limited training data. The FTNB algorithm [14,15] aims to find better estimations for the probability terms used by the NB algorithm. Finding better estimations for the probability terms is particularly important in domains where the training data (labeled data) is limited. The algorithm builds an initial NB classifier and then uses it to determine the misclassified training instances. These misclassified instances are used in the fine-tuning stage to find better estimations of the probability terms responsible for the error. If a training instance, inst, of the form $\;<a_{1},a_{2},\cdots ,a_{m}>$, is misclassified, it means that the predicted class, $c_{predicted}$, has higher probability than the actual class, $c_{actual}$, given the instance’s other attribute values. During the fine-tuning stage, the probability terms involved are modified in such a way that decreases the conditional probability of $c_{predicted}$ given the instance’s other attribute values (i.e., $p\left(c_{predicted}|\;a_{1},a_{2},\cdots ,a_{m}\right)$), and increases the conditional probability of $c_{actual}$ given the instance’s other attribute values (i.e., $p\left(c_{actual}|\;a_{1},a_{2},\cdots ,a_{m}\right))$. The process continues so long as the classification accuracy continues to improve. Algorithm 1 shows the details of the FTNB algorithm. 

The probability terms that need to be decreased are those that are involved in computing $p\left(c_{predicted}|\;a_{1},a_{2},\cdots ,a_{m}\right)$, namely $p\left(c_{predicted}\right)$ and $p\left(a_{i}|c_{predicted}\right)$, where $a_{i}$ is the value of the *i*th attribute of the instance. On the other hand, the probability terms $p\left(c_{actual}\right)$ and $p\left(a_{i}|c_{actual}\right)$, which are involved in computing the $p\left(c_{actual}|\;a_{1},a_{2},\cdots ,a_{m}\right)$, need to be increased. 

Increasing $p\left(c_{actual}\right)$ and decreasing $p\left(c_{predicted}\right)$ give little or no improvement in classification accuracy [14], probably because these two terms are estimated using a large number of instances compared to $p\left(a_{i}|c_{predicted}\right)$ and $p\left(a_{i}|c_{actual}\right)$. Following [14], we do not try to fine-tune these two terms in this work. 

Equations (4) and (5) determine the amount to update $p\left(a_{i}|c_{actual}\right)$ and $p\left(a_{i}|c_{predicted}\right)$, respectively.

(4) $$\delta_{t+1}\left(a_{i},c_{actual}\right)=\eta \cdot \left(\alpha \cdot p\left(max_{i}|c_{actual}\right)-p\left(a_{i}|c_{actual}\right)\right)\cdot error,$$

(5) $$\delta_{t+1}\left(a_{i},c_{predicted}\right)=\eta \cdot \left(\alpha \cdot p\left(a_{i}|c_{predicted}\right)-p\left(min_{i}|c_{predicted}\right)\right)\cdot error,$$

**Algorithm 1 FTNB (training_instances)****phase 1**Use training_instances to estimate the values of each probability term used by the NB algorithm**phase 2**t=0**while** training classification accuracy improves **do** for each training instance, inst, **do**  **let**
$\;c_{actual}$ be the actual class of inst  **let**
$\;c_{predicted}=classify\left(inst\right)$  **if**
$\;c_{predicted}<>\;c_{actual}$  //misclassified instance   **compute** classification error   **for each** attribute value, $a_{i}$, of inst **do**    $let\;p_{t+1}\left(a_{i}|\;c_{actual}\right)=\;p_{t}\left(a_{i}|\;c_{actual}\right)+\delta_{t+1}\left(a_{i},c_{actual}\right)$    $let\;p_{t+1}\left(a_{i}|\;c_{predicted}\right)=\;p_{t}\left(a_{i}|\;c_{predicted}\right)-\delta_{t+1}\left(a_{i},c_{predicted}\right)$   **endfor**  **endif** **endfor** ***let***
t=t+1**endwhile**

The update steps $\delta_{t+1}\left(a_{i},c_{actual}\right)$ and $\delta_{t+1}\left(a_{i},c_{predicted}\right)$ are proportional to the error, which is computed as

(6) $$error=\left|P\left(c_{actual}\right)-P(c_{predicted})\right|,$$

where

(7) $$P\left(c_{o}\right)=\frac{p(c_{o}|inst)}{\sum_{k}^{m}p(c_{k}|inst)},$$

Equation (7) is used to normalize the probabilities.

The update steps are also proportional to a learning rate, η, which is a value between zero and one that is used to decrease the update step. Equation (4), which calculates the update step size for the probability term $p\left(a_{i}|c_{actual}\right)$, is designed so that the update step (the increment) is large for small terms and small for large terms. This explains why the update step is proportional to $\alpha \cdot p\left(max_{i}|c\right)-p\left(a_{i}|c\right)$, where α is a constant and $max_{i}$ is the value of the *i*th attribute with the maximum probability given $c_{actual}$. Equation (5), which computes the decrement for $p\left(a_{i}|c_{predicted}\right)$, ensures that large probability terms are decreased by a large step value while small terms are decreased by a smaller update step; we used $\alpha \cdot p\left(a_{i}|c_{predicted}\right)-p\left(min_{i}|c_{predicted}\right)$, where $min_{i}$ is the value of the ith attribute with the minimum probability, given that $c_{predicted}$. α is a constant (greater than or equal to one), which is used to control the amount of update for $p\left(max_{i}|c\right)$ and $p\left(min_{i}|c_{predicted}\right)$. Setting α to one means these terms gets zero as the update step size. Following [14], we set α to two in all our experiments. Algorithm 1 shows the FTNB algorithm in detail. 

In [27], a fine-tuning method was proposed to fine-tune Bayesian networks. In [28], differential evolution was used to find better estimations for the probability terms used by NB for text classification. In [29], differential evolution was used to find better estimations for the probability terms used in some distance measures, used in instance-based learning [30], such as the VDM [31] and ISCDM [32].

## 3. Bagging NB and the Fine-Tuning Algorithms

In this section, we present the results we obtained by bagging the NB and the FTNB algorithms for text classification. Then, we propose some modifications to the FTNB algorithm to make it less stable and more accurate for text classification, and thus more suitable for bagging.

### 3.1. Bagging the NB and FTNB Algorithms for Text Classification

We conducted several experiments to verify our assumptions and claims, which were: (1) Bagging the NB for text classification does not produce significant improvement, whereas (2) bagging a set of fine-tuned NB classifiers achieves better results. In all experiments, we used 16-benchmark text-classification data sets obtained from the Weka [33] website. Table 1 gives a brief description of the used data sets. All ordinal attributes were discretized using Fayyad et al. [34] supervised discretization method, as implemented in Weka [33]. All of our algorithms were implemented within the Weka framework. Ten-fold cross validation was used in all our experiments. A paired t-test with a confidence level of 95% was used to determine if each difference was statistically significant. 

**Figure 1:**
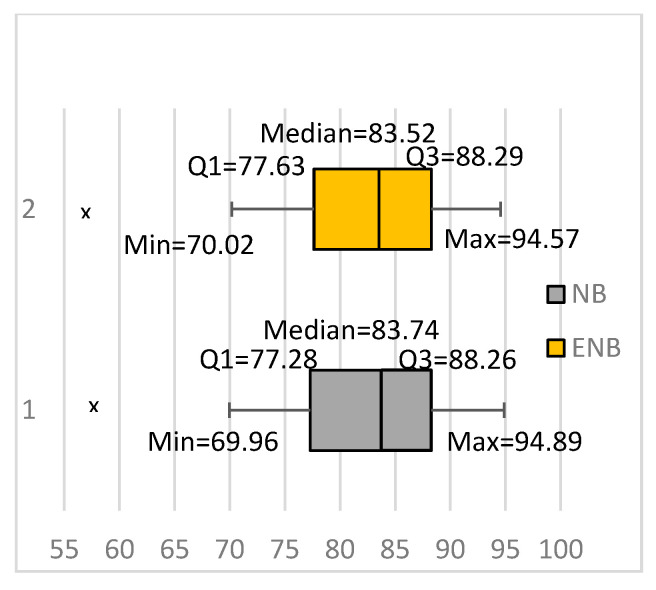
Building ensembles using the naive Bayesian (NB) learning algorithm for text classification. ENB: Ensemble of NB classifiers.

Our results showed that bagging the NB did not really improve the classification accuracy for text classification. Figure 1 summarizes our results as box plots. One box plot shows the results we obtained from bagging an ensemble of 10 NB classifiers, denoted in the figure as ENB. The figure also shows a box plot representing the results we obtained using NB classifiers (trained using all training instances). The figure shows that NB and ENB achieved comparable results. The two methods had very close minimum and maximum values, first quartiles, medians, and third quartiles. They also had close outliers, denoted in the figure using Xs. In fact, the ENB achieved slightly lower average classification accuracy than NB. It achieved an average classification accuracy of 81.96%, whereas NB achieved an 81.98% average accuracy. Moreover, the ensemble achieved significantly better results for one data set and significantly worse results for three data sets. The results show that bagging the NB does not really improve the classification accuracy for text classification.

**Figure 2:**
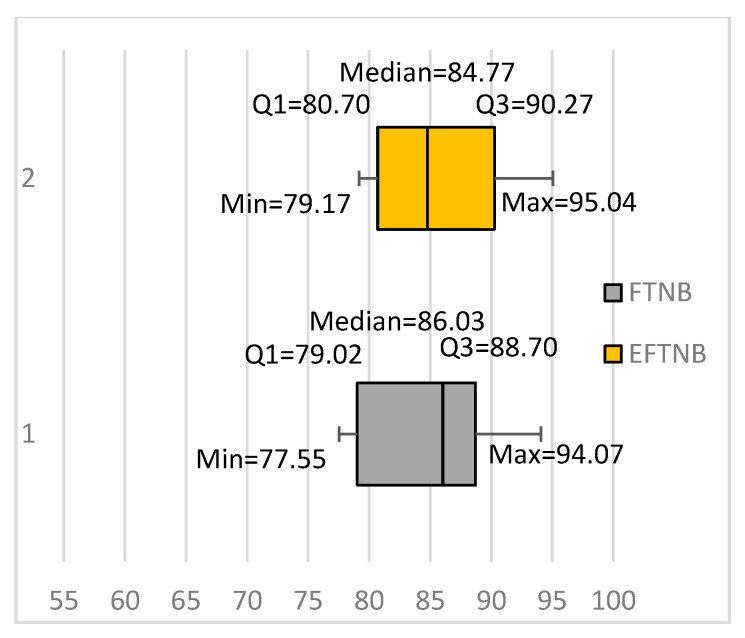
Building ensembles using the fine-tuning NB (FTNB) algorithm classifiers for text classification. EFTNB: Ensemble of FTNB classifiers.

In a similar way we conducted, a set of experiments to compare FTNB classifiers with an ensemble of 10 FTNB classifiers, built using bagging [5]. To ensure more diversity, we also used a random learning rate in the range 0–0.0099 to fine-tune each classifier. The results showed that the ensemble actually achieved better results than both the NB and FTNB algorithms, with an average accuracy of 85.26%. More importantly, the ensemble achieved significantly better results than the FTNB classifier for five data sets and worse results for two data sets. Compared with the NB algorithm, the ensemble achieved significantly better results for eight data sets and significantly worse results for four data sets. Figure 2 summarizes our results as two box plots denoted by EFTNB, for the ensemble of FTNB classifiers, and FTNB for the fine-tuned classifiers. The box plots show that the ensembles had higher values for the minimum, maximum, first quartiles, and third quartiles. The only exception was the median, where FTNB had a higher median than EFTNB. These results support our suspicion that the fine-tuning algorithm would produce diverse classifiers, which makes bagging them as an ensemble of classifiers worthwhile. Without being sufficiently diverse an ensemble of bagged classifiers cannot outperform a base classifier trained using all training instances. 

Our results also showed a substantial increase in the average classification accuracy of the FTNB algorithm compared to the NB algorithm. The average classification accuracy of the FTNB and NB algorithms were 85.14% and 81.98%, respectively. Furthermore, comparing the box plots for NB (Figure 1) and FTNB (Figure 2) showed that the FTNB had higher values for the minimum value, first quartile, median, and third quartile. The only exception was the maximum value, where FTNB had a slightly lower maximum value. Comparing the first quartiles of the two algorithms showed that 75% of the data sets had accuracies above 77.28% and 79.02% using NB and FTNB, respectively. 

However, the FTNB algorithm achieved significantly better results than NB for seven data sets and significantly worse results for five data sets. This indicated that the FTNB algorithm was not well suited for the text classification problem. The average number of the fine-tuning iterations performed by the FTNB was 3.93 iterations. This number was relatively small, which indicated that, for the FTNB algorithm to achieve better results for text classification, the fine-tuning process should be more delicate and the update steps should be smaller. This finding was despite the fact that we used a learning rate of 0.001 in our experiments, which was much smaller, and gave better results, than the learning rate of 0.01 recommended in [14]. The parameter α was set to two, as recommended by [14].

To further improve the results of the fine-tuned classifiers and the ensemble of classifiers, we believe that we need to make the fine-tuning process more gradual (or more delicate) and we need to increase the diversity of the produced classifiers. In the next section, we propose some modifications to the fine-tuning algorithm that makes it more gradual and less stable. By making it less stable, we aim to produce classifiers that are more diverse and thus more suitable for building ensembles of.

### 3.2. A More Gradual Fine-Tuning Algorithm (GFTNB)

As previously discussed, building an ensemble of classifiers is beneficial if the machine-learning algorithm used is unstable enough to produce diverse classifiers. For this reason, we introduce two modifications to the FTNB algorithm that makes it even less stable and thus more likely to produce diverse classifiers. Moreover, the modifications we propose make the weight update process more gradual by making the fine-tuning steps smaller.

#### 3.2.1. Modifying the Update Equations 

We modify the probability update Equations (4) and (5) by replacing each of them with Equation (8).

(8) $$\delta_{t+1}\left(a_{i},c_{actual}\right)=\eta /t\cdot error,$$

where *t* is the iteration number. Equation (8) is different from Equation (4) in two ways. First, it does not use the expression $\left(\alpha \cdot p\left(max_{i}|c_{actual}\right)-p\left(a_{i}|c_{actual}\right)\right)$ because this expression actually condenses the size of the update step and consequently makes the value of the probability terms, in the different classifiers, closer to each other. However, eliminating such subexpression increases the size of the update step, which is good for increasing diversity, but it could decrease the classification accuracy of each constituent classifier. To compensate for this, we use a decaying learning rate by dividing η by the iteration number, *t*, which reduces the size of the update step in later iterations. Replacing Equation (5) with (8) aims to achieve the same result for $\delta_{t+1}\left(a_{i},c_{predicted}\right)$.

Therefore, the modification ensures that we use relatively large update steps at early fine-tuning iterations, and that these steps get smaller at later iterations. This helps to diversify the classifiers by setting them on different paths. At later iterations, the fine-tuning steps become smaller and smaller, which makes the fine-tuning process more gradual.

Table 2 shows the results we obtained by the modified algorithm for the 16-benchmark text-classification data sets compared to the FTNB and NB algorithms. We call the new algorithm the Gradual FTNB (GFTNB) algorithm. We used the 16-benchmark text-classification data sets to compare the classification accuracy of the GFTNB algorithm with the FTNB and NB algorithms. The table shows the results of bagging an ensemble of 10 GFTNB classifiers (EGFTNB) compared with a single GFTNB classifier. Each figure in Table 2 is the average of the 10-fold experiments, as 10-fold cross validation was used in all experiments. The better results are highlighted in bold, and the significantly better results are highlighted in bold and underlined. The last two rows in the table present the number of data sets for which the methods achieved better accuracy and significantly better accuracy.

Our results showed that the GFTNB algorithm outperformed the FTNB algorithm in terms of the average classification accuracy for the 16 text-classification data sets, and in terms of the number of data sets for which it achieved better and significantly better average accuracy. The average accuracy of the GFTNB algorithm was 86.92%, whereas the average accuracy of the FTNB algorithm was 85.14%. The GFTNB algorithm achieved better results than the FTNB algorithm for 14 data sets; seven of them were significantly better results. On the other hand, the FTNB algorithm achieved better results for one data set but that result was not significantly better. The results were also statistically significant according to the Wilcoxon signed rank test at 95% confidence.

However, in terms of the fine-tuning iterations, the GFTNB algorithm required more fine-tuning iterations for most data sets. The average number of fine-tuning iterations for the GFTNB and FTNB algorithms were 5.23 and 3.93, respectively, which indicated that the GFTNB algorithm was actually a more gradual fine-tuning algorithm than the FTNB.

Compared with the NB algorithm, the GFTNB algorithm achieved better results for 12 data sets; 10 of which were significantly better results, whereas NB achieved better results for three data sets, only one of which was a significantly better result. The average accuracy of the NB algorithm for all data sets was 81.98%, which was lower by 4.94% than the average accuracy of the GFTNB algorithm. 

**Figure 3:**
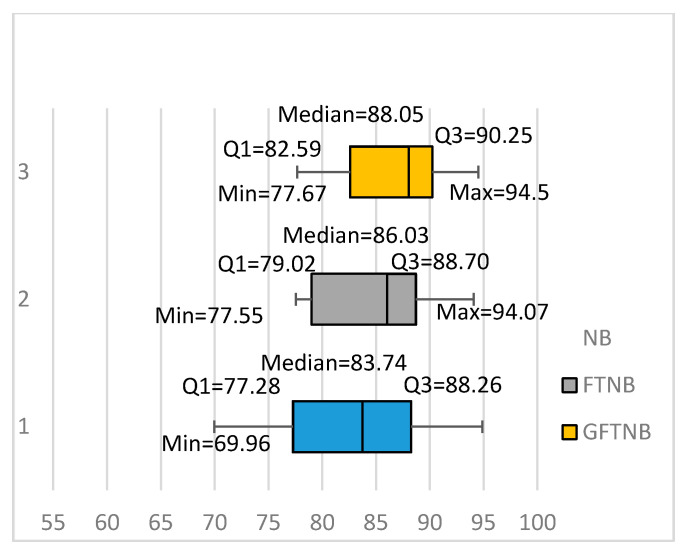
Comparing the FTNB, GFTNB, and NB algorithms.

Figure 3 summarizes our results using a box plot for each of the three algorithms: NB, FTNB, and GFTNB. It can be easily seen that the GFTNB algorithm had a higher minimum value, first quartile, median, and third quartile than the NB and FTNB algorithms. The NB algorithm had a slightly higher maximum value than the two fine-tuning algorithms. Comparing the medians of NB, FTNB, and GFTNB, showed that 50% of that data had an average accuracy above 83.74%, 86.03%, and 88.05%, respectively. Similarly, comparing the third quartiles showed that 25% of the data sets had a classification accuracy above 88.26%, 88.70%, 90.25%, using NB, FTNB, and GFTNB, respectively. All these results indicated that the proposed GFTNB outperformed FTNB and NB for text classification.

**Figure 4:**
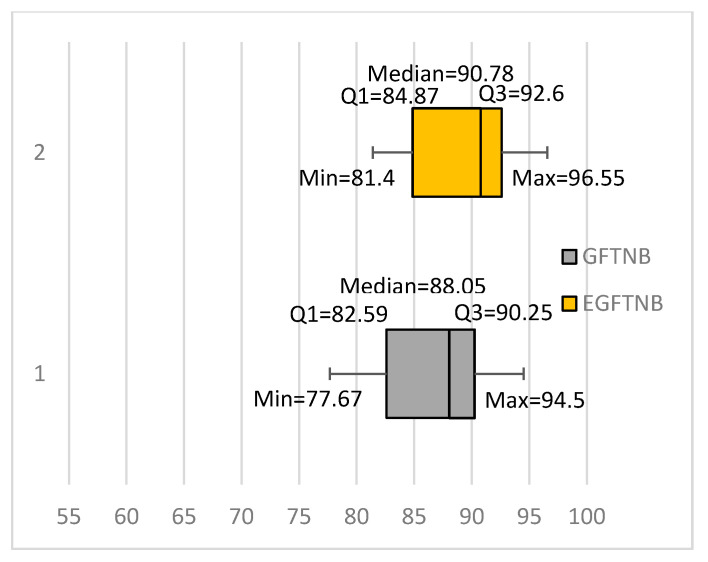
Comparing GFTNB with an ensemble of 10 GFTNB classifiers.

Table 2 also shows the result of an ensemble of 10 GFTNB (EGFTNB) classifiers compared with the GFTNB and NB classifiers. We constructed EGFTNB in the same way we constructed EFTNB. The ensemble was even more accurate than a single GFTNB classifier, trained using all training instances, for 13 data sets, 11 of which were significantly better results, and less accurate for three data sets, but none of which were significantly worse results. The average accuracy of the ensemble was 88.67%, which was higher by 1.75% than the average accuracy of GFTNB. Recall that EFTNB (an ensemble of 10 FTNB classifiers) achieved better results than FTNB for 10 data sets, only five of which were significantly better results. Moreover, EFTNB achieved worse results than FTNB for six data sets, two of which were significantly worse results. Comparing the box plots of EGFTNB and GFTNB (see Figure 4), showed that the ensemble had a higher minimum value, first quartile, median, third quartile, and maximum value, by more than 2% than the corresponding values of GFTNB. Comparing EGFTNB with NB showed that the EGFTNB outperformed NB for 15 data sets, of which 12 were significantly better results, whereas NB outperformed EGFTNB for one data set and that result was statistically significant (see Table 2). We compared the results of EFTNB and EGFTNB using Wilcoxon signed-rank test and found out that EGFTNB was significantly better at 99% confidence. 

These results show that the GFTNB algorithm produced more suitable classifiers for bagging than the classifiers produced by FTNB. The fact that an ensemble of GFTNB classifiers produced far better results than a single GFTNB classifier (trained using all training instances) indicates that the GFTNB algorithm produced a more diverse ensemble of classifiers.

It is worth mentioning that we also constructed an ensemble of 20 GFTNB classifiers (EGFTNB-20). EGFTNB-20 achieved an average accuracy of 89.18%, which was an increase of 0.51% compared to the average accuracy of EGFTNB (10 classifiers). However, the number of data sets for which EGFTNB-20 achieved better and significantly better results than the NB classifier remained the same as those of the EGFTNB. Fine-tuning EGFTNB-20 required an average of 97.36 fine-tuning iterations, whereas EGFTNB-10 required an average of 49.29 iterations. 

#### 3.2.2. Modifying the Termination Condition

To further increase the diversity among the constituent classifiers, we modified the fine-tuning termination condition. Each constituent classifier was fine-tuned for a random number of iterations, between 5 and 15, and the probability values that gave the best training accuracy were taken as the result of the fine-tuning process. 

**Figure 5:**
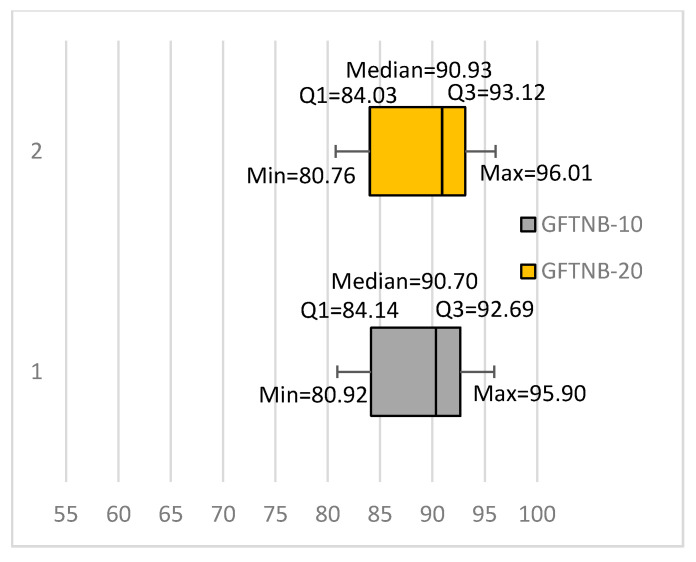
An ensemble of 10 (GFTNB-10) and 20 classifiers (GFTNB-20) after modifying the termination condition.

We constructed two ensembles of 10 classifiers and 20 classifiers. We called them GFTNB-10 and GFTNB-20, respectively. The latest modification increased the average classification accuracy of the ensemble of 10 classifiers from 88.67% (the average accuracy of EGFTNB) to 88.83%, whereas the ensemble of 20 classifiers increased the average to 89.17%. Moreover, the GFTNB-10 outperformed the NB classifier for 15 data sets, 12 of which were statistically significant, whereas the NB classifier outperformed the GFTNB-10 for only one data set, but not in a statistically significant way. The performance of the GFTNB-20 was even better; it outperformed the NB classifier for 15 data sets, 13 of which were statistically significant, whereas NB outperformed the GFTNB-20 for only one data set, but not in a statistically significant way. This modification increased the number of fine-tuning iterations. The GFTNB-10 required, on average, twice the number of iterations to fine-tune than the EGFTNB. Obviously, fine-tuning the twenty classifiers in the GFTNB-20 required even more iterations; the GFTNB-20 required, on average, 190.79 iterations. Figure 5 shows the box plots diagrams for GFTNB-10 and GFTNB-20. 

Table 3 summarizes the performance of each algorithm compared with the NB algorithm, in terms of the number of the data sets for which the algorithm achieved significantly better results (wins), the number of data sets for which no algorithm achieved significantly better results (ties), and the number of data sets for which the NB algorithm achieved significantly better results (losses). Table 3 also shows the average improvement in accuracy achieved by each algorithm compared to the average of the NB algorithm (i.e., the difference in average). 

## 4. Conclusions

This work addresses the issue of constructing an ensemble of NB classifiers for text classification using the bagging method [5]. It empirically shows that an ensemble of NB classifiers achieves little or no improvement in classification accuracy. However, an ensemble of fine-tuned NB classifiers achieves significantly more accurate results for many data sets. This study also proposes a more accurate fine-tuning algorithm for text classification, and empirically shows that this algorithm is more suitable for building an ensemble of fine-tuned classifiers than the original fine-tuning algorithm, using the bagging method. This work uses the bagging method for ensemble construction, combined with parameter modification (learning rate and the number of fine-tuning iterations) to increase diversity. Future work may investigate using boosting as a method for constructing an ensemble of fine-tuned NB classifiers for text classification. Fine-tuning other variants of NB for text classification, such as multinomial NB [35], and constructing ensembles of such fine-tuned classifiers might be an interesting future research area. 

## Figures and Tables

**Table 1:** A description of the data sets used in the experiments.

Dataset	#Documents	#Words	#Classes
**Fbis**	2463	2000	17
**La1s**	3204	13,196	6
**La2s**	3075	12,433	6
**Oh0**	1003	3182	10
**Oh10**	1050	3238	10
**Oh15**	913	3100	10
**Oh5**	918	3012	10
**Re0**	1657	3758	25
**Re1**	1504	2886	13
**Tr11**	414	6429	9
**Tr12**	313	5804	8
**Tr21**	336	7902	6
**Tr31**	927	10,128	7
**Tr41**	878	7454	10
**Tr45**	690	8261	10
**Wap**	1560	8460	20

**Table 2:** The results ofGFTNB and EGFTNB vs. NB and FTNB.

Data Set	FTNB vs. GFTNB		#Iterations		NB vs. GFTNB		GFTNB vs. EGFTNB		#Iterations	NB vs. EGFTNB	
	FTNB%	GFTNB%	FTNB	GFTNB	NB%	GFTNB%	GFTNB%	EGFTNB%	EGFTNB	NB%	EGFTNB%
Fbis.wc	77.55	**77.67**	10.3	13.1	69.96	**77.67**	77.67	**81.20**	68.1	69.96	**81.20**
La1s.wc	86.33	**89.79**	2.8	5	86.55	**89.79**	89.79	**91.26**	57.1	86.55	**91.26**
La2s.wc	84.26	**89.63**	3.1	6.1	87.48	**89.63**	89.63	**91.71**	53.7	87.48	**91.71**
Oh0.wc	89.63	**91.63**	3.3	4.1	91.43	**91.63**	91.63	**93.12**	46.3	91.43	**93.12**
Oh5.wc	85.73	**88.13**	3.2	5.9	84.42	**88.13**	88.13	**89.98**	50.6	84.42	**89.98**
Oh10.wc	77.62	**82.10**	2.8	5.7	**83.05**	82.10	82.10	**83.62**	50.1	83.05	**83.62**
Oh15.wc	83.24	**85.21**	2.6	4.9	**85.43**	85.21	85.21	**86.09**	52.3	85.43	**86.09**
Re0.wc	79.06	**80.19**	6.7	5	74.47	**80.19**	80.19	**82.71**	54.3	74.47	**82.71**
Re1.wc	78.76	**79.72**	3	3.5	77.37	**79.72**	79.72	**83.40**	47	77.37	**83.40**
Tr11.wc	86.96	**87.92**	4	4.4	77.29	**87.92**	**87.92**	87.68	45.3	77.29	**87.68**
Tr12.wc	92.33	**92.97**	2.9	3	**94.89**	92.97	**92.97**	92.65	39.5	**94.89**	92.65
Tr21.wc	88.39	88.39	4.1	2.6	58.04	**88.39**	88.39	**91.67**	41.5	58.04	**91.67**
Tr31.wc	94.07	**94.50**	5.3	5.6	90.61	**94.50**	94.50	**95.90**	40	90.61	**95.90**
Tr41.wc	91.23	**92.14**	2.6	4.2	92.14	92.14	92.14	**92.94**	43.6	92.14	**92.94**
Tr45.wc	**88.12**	87.97	3.1	5.1	77.25	**87.97**	87.97	**93.04**	44.3	77.25	**93.04**
Wap.wc	78.91	**82.76**	3.1	5.4	81.35	**82.76**	**82.76**	81.67	54.9	81.35	**81.67**
**average**	**85.14**	**86.92**	**3.93**	**5.23**	**81.98**	**86.92**	**86.92**	**88.67**	**49.29**	**81.98**	**88.67**
**#better**	**1**	**14**			**3**	**12**	**3**	**13**		**1**	**15**
**#Sig Better**	**0**	**7**			**1**	**10**	**0**	**11**		**1**	**12**

**Table 3:** The performance of each algorithm compared to NB.

Algorithm	Average Higher by	Wins	Ties	Losses
ENB	−0.02%	1	12	3
FTNB	3.16%	7	4	5
EFTNB	3.28%	5	9	2
GFTNB	4.92%	10	5	1
EGFTNB	6.69%	12	3	1
GFTNB-10	6.85%	12	4	0
GFTNB-20	7.19%	13	3	0
